# Sociodemographic and clinical characteristics, causal factors and evolution of a group of patients with chronic urticaria-angioedema

**DOI:** 10.1590/S1516-31802007000500006

**Published:** 2007-09-02

**Authors:** Kunie Iabuki Rabello Coelho, Ivete Dalben, Joel Carlos Lastória, Luciana Patrícia Fernandes Abbade

**Keywords:** Urticaria, Angioneurotic edema, Socioeconomic factors, Infection, Epidemiology, Urticária, Edema angioneurótico, Fatores socioeconômicos, Infecção, Epidemiologia

## Abstract

**CONTEXT AND OBJECTIVE:**

Chronic urticaria-angioedema is a common, multiple-cause complaint. The aim was to investigate the sociodemographic and clinical characteristics, causal and aggravating factors and evolution of urticaria-angioedema.

**DESIGN AND SETTING:**

This was a descriptive prospective study carried out at the Dermatology outpatient clinic of Faculdade de Medicina de Botucatu, Universidade Estadual Paulista (Unesp).

**METHODS:**

A total of 125 patients with chronic urticaria-angioedema were evaluated to obtain sociodemographic data, anamnesis, dermatological and general clinical data and laboratory data, emphasizing causal and aggravating factors and complaint evolution.

**RESULTS:**

Chronic urticaria-angioedema occurred mainly in females (mean age: 35 years), but also in men (mean age: 32 years). White color and living in urban areas also predominated. There was no preferential time for symptoms to appear, and nighttime was the most commonly reported time for clinical worsening. Around half of the patients had urticaria associated with angioedema. There were no associated factors in most of the cases, and stress was the most commonly reported aggravating factor. The cause was ascertained in 37.6% of our cases. The mean duration of follow-up was 11.7 months. Around 60% of the patients evolved with the problem under control, 32% improved, 9% had no change in dermatological condition and only one patient worsened.

**CONCLUSIONS:**

Chronic urticaria-angioedema was more common among middle-aged women. It is a long-term disease, and its cause was explained in about one-third of the patients. Half of the patients presented disease control after treatment lasting an average of approximately one year.

## RESUMO

**CONTEXTO E OBJETIVO:**

Urticária-angioedema crônico é enfermidade freqüente, complexa e multicausal. O objetivo foi estudar as características sociodemográficas, clínicas, os fatores causais, agravantes e a evolução da enfermidade.

**TIPO DE ESTUDO E LOCAL:**

Descritivo e prospectivo, realizado no ambulatório de Dermatologia da Faculdade de Medicina de Botucatu, Universidade Estadual Paulista (Unesp).

**MÉTODOS:**

Foram avaliados pacientes com diagnóstico de urticária-angioedema crônico através de dados sociodemográficos, anamnese, exames dermatológico, clínico e laboratorial, com ênfase nos fatores causais, agravantes e na evolução da enfermidade.

**RESULTADOS:**

125 pacientes foram incluídos, 95 mulheres e 30 homens. Predominaram mulheres de 30 a 40 anos e homens de 10 a 20 anos. A idade média foi de 35 anos para as mulheres e 32 anos para os homens. Predominaram pacientes de raça branca, residentes em zona urbana e casados. O tempo médio de doença foi de 45,6 meses e de cada lesão foi de 5,6 horas. A metade dos casos tinha surtos diariamente e associação de urticária com angioedema. Não houve horário preferencial de aparecimento dos surtos, mas o noturno foi o horário de piora mais citado. A causa foi esclarecida em 37,6%, predominando as infecções. O estresse foi o agravante mais referido. O tempo médio de acompanhamento foi de 11,7 meses e 60% evoluíram para o controle, 32% melhoraram, 9% mantiveram-se inalterados e um caso piorou.

**CONCLUSÕES:**

Urticária-angioedema ocorreu mais em mulheres de meia-idade. A causa foi esclarecida em um terço dos pacientes e metade deles teve controle da doença em aproximadamente um ano.

## INTRODUCTION

Urticaria-angioedema is a frequent complaint, characterized by maculae, papules and edematous, pruriginous erythematous plaques that suddenly appear and are short lived, spontaneously disappearing in minutes, hours or days.^1–5^ The lesions are called wheals and appear in varying number, shape, size and location on the tegument (urticaria). They are often accompanied by edema on the eyelids, lips, tongue, genitals and palm and sole regions (angioedema). When they affect the respiratory, cardiovascular or gastrointestinal systems, it is called anaphylaxis.^3,5–7^

Episodes may occur daily or at irregular intervals,^1,2,4^ can recur for an indefinite time^3^ and are considered chronic when the clinical picture persists for six weeks or more.^1,3,4,7–15^ Urticaria-angioedema can attack at any age and particularly affects middle-aged women.^1–4,6,8,10,12–14,16,17^ It is estimated that between 12% and 25% of the general population have already had at least one urticaria-angioedema episode^16,17^ and that the prevalence among dermatological patients is around 1.85%.^8,18^ The illness may have spontaneous resolution, but by detecting the cause(s) and aggravating factor(s) it can be controlled.^4,19–21^

## OBJECTIVE

Considering its high prevalence in our service, the difficulty and complexity in establishing its cause(s), and the many different aspects of the clinical presentation of urticaria-angioedema, the general objective of this study was to recognize and describe the sociodemographic and clinical characteristics, causal and aggravating factors, duration of follow-up and evolution of this complaint.

## METHOD

This descriptive prospective study was carried out by analyzing all the patients who came to the allergy outpatient clinic of the Department of Dermatology, Hospital das Clínicas, Faculdade de Medicina de Botucatu (FMB), Universidade Estadual Paulista (Unesp), and presented a clinical history compatible with chronic urticaria-angioedema, from August 1990 to January 1998. This project was approved by the Research Ethics Committee of FMB/Unesp, and consent was obtained from each patient.

Cases of chronic urticaria-angioedema were defined as such when their duration was six weeks or more, in line with most data in the literature.^1,3,4,7–15^ Patients with acute or hereditary urticaria-angioedema were excluded from this study.

A protocol was designed for this study, which included sociodemographic and clinical variables, causal factors and disease evolution. The sociodemographic variables included age, gender, race, marital status, occupation and origin. The places where the patients lived were characterized as rural or urban areas. The clinical variables included length of time since symptom onset, outbreak or episode frequency, location and duration of lesions, preferred appearance time and any clinical worsening of the condition. Aggravating factors and associated symptoms were also evaluated; these included fever, arthralgia, chronic headache and abdominal pain.

Causal factors were obtained through anamnesis, dermatological and general physical examination and laboratory tests such as complete blood count with differential analysis and erythrocyte sedimentation rate (ESR), urine analysis and stool examination. Standard blood biochemical analyses were performed to obtain fasting glycemia levels. Hepatic, renal and thyroid function tests were performed. Inflammatory markers, serology for autoimmune diseases, toxoplasmosis, mononucleosis, hepatitis B and C and total immunoglobulin E (IgE) were investigated. Facial sinus tests, chest X rays, upper digestive tract endoscopy, skin lesion biopsies and prick tests were also performed. Direct mycological examinations were performed when indicated by anamnesis or physical and dermatological examination. Additional provocation tests such as the ice cube test, water test, physical exercise, medicine suspension, food exclusion followed by reintroduction and the prick test were included. Alcohol, heat, fever, medication, physical exercise, emotional stress, local pressure, sweating and bathing were considered to be aggravating factors.

The disease evolution was determined by evaluating the duration of outpatient attendance (months), any suspension or treatment of suspected cause, any use of symptomatic medication and the patient's clinical situation at last visit (whether improved, worse, stable or without complaint).

A database was constructed using the Epi-Info^TM^ (version 6.04) statistics software and statistical analysis was performed using the Statistical Package for the Social Sciences (SPSS, version 10.01). The data were transcribed by means of a previously tested semi-structured instrument and were coded, digitized and analyzed. The frequencies between different variables were established using the chi-squared or Fisher exact tests, as needed. Differences between means were evaluated by the Student t test. All tests were bicaudal and the significance level was 5% (p < 0.05).

## RESULTS

The study included 125 patients with chronic urticaria-angioedema; 95 (76%) were female and 30 (24 %) male, giving a male/female ratio of 1:3.

The age of occurrence ranged from 3 to 77 years; 82.4% of the cases were between 10 and 50 years old, 1.6% were under 10 years old and 16% were over 50 years old. The highest number of cases occurred between 30 and 40 years old (25.6%), followed by 10 to 20 years (20.0%), 20 to 30 years (19.2%), and 40 to 50 years (17.6%). There was a predominance of females between 30 and 40 years (28.4%), and males between 10 and 20 years (30%). The mean age for the women was 35.3 (± 15.4) years and for men it was 32.1 (± 17.6) years (p = 0.3). White skin color (94.4%) and married status (59.2%) predominated (Table 1).

**Table 1.** Relative and absolute distribution of the patients with chronic urticaria- angioedema, according to age, race, marital status and gender

Variable		Gender		
		Female	Male	Total
		n = 95 (100%)	n = 30 (100%)	n = 125 (100%)
**Age**	0 to 10	1 (1.1)	1 (3.3)	2 (1.6)
	10 to 20	16 (16.8)	9 (30.0)	25 (20.0)
	20 to 30	18 (18.9)	6 (20.0)	24 (19.2)
	30 to 40	27 (28.4)	5 (16.7)	32 (25.6)
	40 to 50	19 (20.0)	3 (10.0)	22 (17.6)
	50 and over	14 (14.7)	6 (20.0)	20 (16.0)
	Mean age	35.3	32.1	38.7
**Race**	White	90 (44.7)	28 (93.3)	118 (94.4)
	Non-white	5 (5.3)	2 (6.7)	7 (5.6)
**Marital status**	Single	36 (37.9)	15 (50)	51 (40.8)
	Married	59 (62.1)	15 (50)	74 (59.2)

Among the patients’ occupations, 66 (52.8%) were classified as inactive (32% housewives, 15.2% students and 5.6% unemployed or retired). There was no predominance of occupational group within the 47.25% who were classified as active.

With regard to origin, 41.6% were from Botucatu city, and the rest were from other regions of the State of São Paulo; 89.6% lived in an urban zone, 9.6% in a rural zone, and 0.8% in an intermediate rural/urban zone.

The length of time since onset ranged from two to 360 months, with a mean of 45.26 months. Daily reappearance was detected in 52.8%, weekly in 4%, fortnightly in 3.2%, monthly in 0.8% and irregular in 39.2%.

An association between urticaria and angioedema occurred in 50.4%, while 48.8% had urticaria alone and 0.8% had angioedema alone.

The mean duration of each lesion was 5.6 hours and it ranged from a few minutes to 48 hours in 86.4% of the cases. The most commonly reported interval was between one and six hours (28.8%), followed by the interval between a few minutes and one hour (25.6%), six to 12 hours (16%), 12 to 24 hours (13.6%) and 24 to 48 hours (2.4%). About 13% of the patients did not respond to this item.

There was no preferential time for the appearance of the lesions in 94.4% of cases, but 2.4% reported preferential appearance in the morning, 1.6% in the afternoon and 1.6% at night. However, when patients already had the lesions, 20.8% reported worsening at night, 14.4% in the afternoon, 11.2% in the morning and 0.8% both in the morning and at night. There was no time of worsening according to 52.8% of the patients, and around 5% did not respond to this item.

Urticaria-angioedema was not accompanied by any other symptom in 90.4%; 4% reported arthralgia and 2.4% chronic headache.

Worsening factors were reported by 84.0% of the cases. Stress was reported by 15.2%, followed by local pressure by 7.2% and medications by 7.2%. Food, heat, cold and alcohol consumption were reported by 4.4%, and about 50% reported two or more concomitant aggravating factors.

Prior personal allergies were reported by 43.2% and prior allergies among some members of the family by 44.8%. The prick test was performed with a standard inhalant set for all patients; the results were positive in 6.7%.

There was no causal relationship with domestic activity for 93.6%, environmental substances for 74.8%, working environment for 90.4%, leisure activities for 91.2%, cosmetics use for 99.2% and alcohol and cigarette use for 98.4%. Medication was not reported as the cause for 75.2% but was confirmed by 4%; 20.8% did not respond to this item. Infectious foci were not reported by 85.6% and the rest of the patients did not respond to this item. Foods were not reported as a cause by 75.2% but were reported as a cause by 7.2%, while 17.6% did not respond. Afterwards, only one patient was found to present confirmed food urticaria after undertaking an exclusion diet and then being re-exposed to the food.

In the dermatological examination, 37.6% were found to have another dermatological complaint such as dermatophyte fungal infections (22.4%), acne (5.6%), cutaneous tinea versicolor or xerosis (2.4%), seborrheic dermatitis (2.4%) and warts, varices and contact dermatitis (1.6% each). One case of each of the following were also detected: chronic eczematous inflammation, psoriasis, atopic dermatitis, stasis dermatitis, scabies, pityriasis alba, acquired immune deficiency syndrome, dyshidrosis, intertrigo and tylosis (0.8% each).

By correlating the anamnesis with the dermatological, physical and laboratory tests, 16 patients (12.8%) were found to have bacterial or fungal infection of the skin, teeth, upper airways or genitourinary areas.

Blood and stool tests were performed on 104 of the patients. 24% presented abnormalities in blood tests (increased ESR in 8.7%, eosinophilia in 7.7%, anemia in 3.8%, and anemia and eosinophilia in 1.9%). Stool tests were positive for six patients (5.7%), with helminths in four (3.8%) and protozoa in two (1.9%).

Urine analysis was performed in 105 cases (84%) and only 4.8% presented abnormalities.

The causal hypotheses are listed in Table 2. The cause of chronic urticaria-angioedema was clarified in 37.6% of cases. The predominant causes were infection (in 12.8% of the cases), inhalants (6.7%) and medication (4%). Cholinergic causes, local pressure and intestinal parasitosis occurred in 3.2% each, followed by thyropathy in 2.4% of cases. The least common causes were foods, cold and urticarial vasculitis (0.8% each).

**Table 2.** Distribution of the patients according to causal hypotheses for urti- caria-angiodema

Cause	n = 125 (100%)
Not ascertained*	78 (62.4)
Infection†	16 (12.8)
Inhalant	8 (6.7)
Medication	5 (4.0)
Cholinergic	4 (3.2)
Local pressure	4 (3.2)
Intestinal parasitosis	4 (3.2)
Thyropathy	3 (2.4)
Food‡	1 (0.8)
Cold	1 (0.8)
Urticarial vasculitis	1 (0.8)

* *p = 0.02;*

† *p = 0.001;*

‡ *p = 0.009.*

Twenty-four (19.2%) out of the 125 patients came only for the first consultation and their evolution was therefore unknown. Of the 101 who returned regularly, 58.4% had their condition controlled, 31.7% improved, 8.9% were unchanged, and only one worsened in relation to first consultation (Table 3).

**Table 3.** Distribution of the patients according to their evolution, as observed at the last consultation

Evolution	n = 125 (100%)
Under control	59 (58.4)
Improved	32 (31.7)
Unchanged	9 (8.9)
Worse	1 (0.8)
Unknown	24 (19.2)

The follow-up ranged from one day to 87.37 months (mean: 11.7 months). The mean length of follow-up in our study was 11.7 months, and the range was from one day to 85.37 months. Over the course of the follow-up, 58.4% evolved to achieving disease control, 31.7% improved and 8.9% remained unchanged. Only one patient reported worsening. It is important to emphasize that 19.2% of the patients attended only the first consultation.

## DISCUSSION

This study demonstrated that urticaria angioedema occurs in all age groups, with predominance in females (male/female ratio of 1:3), white patients and the 20 to 50-year age group, which was similar to some authors’ findings.^22–24^ Other authors found male/female ratios of 1:2^8,15,17,25–27^ and 1:1.^9,28^ Only one study reported an inverse ratio, i.e. male predominance (2:1).^18^

In our study, the predominance among females was between the ages of 30 and 40 years, as reported in the literature.^6,8,9,22,25,26^ Among males, it was between 10 and 30 years, differing from Helgreen & Hersle, who reported predominance among males between 30 and 40 years old.^8^

It is difficult to compare and discuss our sociodemographic data with the reports in the literature because of the lack of standardization. There is a tendency towards increased numbers of cases among patients over 15 years of age, and particularly between the ages of 20 and 40 years, both in the literature and in this study.

The patients’ mean age in our study was similar to what was found by several authors^15,17,27,29,30^ (35.3 years for women and 32.1 years for men), and the mean ages for men and women were similar (p = 0.3). However, these were higher than the 24 years and 7 months found by Nizami & Baboo.^25^

There was no prevalence according to occupational group in the present study, which is in agreement with the literature.^8^ Most of the patients were married and from urban areas.

Complaint duration was very variable (two to 360 months; mean 45.26 months). There are reports in the literature of complaints lasting for 2.5 months,^18^ 15 months,^17^ 24 to 48 months^26^ and 52 months.^27^

Daily recurrences occurred in 52% of our patients, which was less than the 62% reported by some authors,^9^ and higher than the 44% reported by others.^26^

The frequency of associated urticaria and angioedema (50.4%) was similar to the findings of Champion et al,^6^ while higher than those of some authors,^27,29^ and lower than others.^9,18,26^ Angioedema without urticaria (0.8%) was less frequent than reported in the literature.^6,9,25,29^

Individual lesion duration ranged from a few minutes to 24 hours (mean: 5.6 hours) in nearly all cases. This also highlights the fugacity of the lesions, in keeping with the correct definition of the disease. We would like to emphasize that we did not find any quantifying data in the literature with any similarities to the data on duration presented in our study. Moreover, 13% of the patients did not know how to report the duration of each lesion because they did not understand the difference between outbreak duration and individual lesion duration.

No preferential time of the day for the complaint to appear was found in our study, which is in agreement with other authors.^9,13,26,27^

Arthralgia, chronic headache, fever and abdominal pain were absent in most of our cases. The frequency of association with arthralgia was similar to some authors’ findings^18,26^ and significantly lower than in other studies.^9,11^ This was probably due to lack of standardization of the concept of arthralgia among researchers and patients’ difficulties in characterizing it. The frequency of chronic headache (2.4% of cases) was less than the 7% found by Juhlin.^26^ Abdominal pain and fever were reported in the literature^9,18,26^ but were not found in our study.

Stress was the most commonly reported worsening factor among our patients (15%); in the literature, the frequency of this factor ranged from 7%^26^ to 42%.^9^ The emotional factor should be carefully analyzed on its own, as proposed by many authors.^6,9,25–27,29^ Physical agents (pressure, site, sun, physical exercise, heat and cold) worsened the clinical condition in 10.4% of the cases, which was a greater number than reported by Miller et al^9^ and markedly less than the 50% found by Sibbald et al.^27^

The cause was ascertained in 37.6% of our cases. The proportions of cases with ascertained causes in the literature were 17%,^9^ 18%,^11^ 21%,^6,12^ 29%,^6^ 31%^6,27^ and 44%.^15,17^ Bacterial infection of the oral cavity, genitourinary areas and facial sinuses, and fungal infections, were the most common causes found in our patients (12.8%) and these findings were similar to the results reported by Martínez Pichardo and Abdo Rodrigues.^22^ Physical agents were the cause in 7.2% of the cases, which was similar to the findings of Small et al.,^11^ higher than those of Martínez Pichardo and Abdo Rodrigues^22^ and lower than other authors.^15,17,27^ Although previous allergies could indicate an association with atopic causes and inhalants,^9,21,25,27^ this on its own does not confirm inhalants as a cause of chronic urticaria-angioedema, and a carefully conducted population-based study would be needed to clarify this. Inhalants as a causal factor were seen in 6.7% of our cases; we sought help from other medical professionals (otorhinolaryngologists and pneumologists) and complementary examinations to confirm them. Medications were the cause in 4.8% of the cases, and these needed only the anamnesis for confirmation. This is similar to what was found by several authors;^22,25^ higher than the 1.2% found by Sibbald et al.,^27^ and less then the 9.1% found by Kozel et al.^17^ Intestinal parasitosis (3.2%) was an important finding among our patients and must always be tested for; its incidence was lower than reported by Martínez Pichardo and Abdo Rodrigues.^22^ The frequency of thyropathy (2.4%) was practically the same as found in the literature.^11^ Food causes were less frequent than reported by others, whose findings ranged from 2.3%^22^ and 6%^9,17,27^ to 30%.^26^ Urticarial vasculitis was rare (0.8%) and less than the 3% found by Small et al.^11^

Data from this study and the literature^8,9,17,18,26,27^ show that anamnesis is a very important criterion for cause indication, and that dermatological and physical examination and the more accessible laboratory tests, such as complete blood count differential analysis, erythrocyte sedimentation rate, urinalysis and stool test must be taken into account in evaluating urticaria-angioedema patients, since they help to detect subclinical infection causes. Other clinical and patient history examinations must also be carried out.

## CONCLUSION

It was possible to conclude that chronic urticaria-angioedema occurred mainly among middle-aged women, individuals with white skin color, people living in urban areas and married patients, regardless of their occupation. The average duration of the disease was long and about half of the patients presented an association between urticaria and angioedema and daily relapse. Angioedema without urticaria was rare. Stress was the main factor in the worsening of this disease and the cause was explained in around one-third of the patients. Bacterial infection was the main cause, followed by physical agents. Food was hardly ever related to the cause. More than half of the patients achieved disease control over an average time of approximately one year of follow-up. The anamnesis, follow-up and routine laboratory tests were the most important procedures for studying the causes.
